# *α*-Adducin Gly460Trp Gene Mutation and Essential Hypertension in a Chinese Population: A Meta-Analysis including 10960 Subjects

**DOI:** 10.1371/journal.pone.0030214

**Published:** 2012-01-17

**Authors:** Yan-yan Li

**Affiliations:** Department of Geriatrics, First Affiliated Hospital of Nanjing Medical University, Nanjing, China; Mayo Clinic, United States of America

## Abstract

**Background:**

The *α-adducin* Gly460Trp (G460W) gene polymorphism may be associated with susceptibility to essential hypertension (EH), but this relationship remains controversial. In an attempt to resolve this issue, we conducted a meta-analysis.

**Methods:**

Twenty-three separated studies involving 5939 EH patients and 5021 controls were retrieved and analyzed. Four ethnicities were included: Han, Kazakh, Mongolian, and She. Eighteen studies with 5087 EH patients and 4183 controls were included in the Han subgroup. Three studies with 636 EH patients and 462 controls were included in the Kazakh subgroup. The Mongolian subgroup was represented by only one study with 100 EH patients and 50 controls; similarly, only one study with 116 EH patients and 326 controls was available for the She subgroup. The pooled and ethnic group odds ratios (ORs) along with the corresponding 95% confidence intervals (95% CI) were assessed using a random effects model.

**Results:**

There was a significant association between the *α-adducin* G460W gene polymorphism and EH in the pooled Chinese population under both an allelic genetic model (OR: 1.12, 95% CI: 1.04–1.20, P = 0.002) and a recessive genetic model (OR: 1.40, 95% CI: 1.16–1.70, P = 0.0005). In contrast, no significant association between the *α-adducin* G460W gene polymorphism and EH was observed in the dominant genetic model (OR: 0.88, 95% CI: 0.72–1.09, P = 0.24). In stratified analysis by ethnicity, significantly increased risk was detected in the Han subgroup under an allelic genetic model (OR: 1.13, 95% CI: 1.04–1.23, P = 0.003) and a recessive genetic model (OR: 1.43, 95% CI: 1.17–1.75, P = 0.0006).

**Conclusions:**

In a Chinese population of mixed ethnicity, the *α-adducin* G460W gene polymorphism was linked to EH susceptibility, most strongly in Han Chinese.

## Introduction

Essential hypertension (EH) is one of the primary causes of cardiovascular diseases. It is a polygenic hereditary disease with a complex pathogenesis that is strongly influenced by environmental and lifestyle factors. Previous studies have indicated that abnormal renal membrane ion transport might play a key role in the onset of EH [1]. There is now a general consensus that the increase in circulating blood volume due to increased renal sodium reabsorption is a major pathogenic factor leading to EH. Adducin is a cytoskeleton protein composed of two subunits (one α-subunit and either a β- or γ-subunit). Adducin has been implicated in the construction and maintenance of the cytolemma framework. In addition, adducin regulates cellular signal transduction and cytolemma ion transport by modulating the surface expression of multiple transporters and pumps, including the Na^+^-H^+^ exchanger, Na^+^-K^+^-Cl^−^ cotransporter, and the sodium pump [2]. Regulation of renal Na^+^ transport by the adducin protein suggests that allelic variants of *adducin* may contribute to EH.

The human *α-adducin* gene, located on 4p16.3, is composed of 16 exons. In 1997, Cusi et al first reported a missense point mutation substituting thymine (T) for guanine (G) at position 614 of the 10^th^ exon, resulting in an expressed adducin α subunit with Trp (W) in place of the wild type Gly (G) at amino acid 460. In 2004, Beeks et al found that this *α-adducin* 460W mutant markedly influenced renal haemodynamics and function. The evident drop in effective renal blood flow and glomerular filtration rate (GFR) in the WW genotype led to excess water and sodium retention, even when patients maintained a low salt diet [3]. It was suggested that the (G→W) alteration of α-subunit might affect protein assembly or stability, leading to changes in submembrane cytoskeleton structure, increased sodium reabsorption by the proximal renal tubules, and reduced GFR. Enhanced water and sodium retention could increase circulating blood flow volume and cause high BP. It remains to be established, however, if the *α-adducin* G460W gene polymorphism does indeed increase susceptibility to a salt-sensitive form of hypertension [4].

In China, hypertensive patients have three features: salt-sensitivity, great BP fluctuation, and high stroke morbidity. Approximately 60% of Chinese hypertensives are salt-sensitive (meaning BP would be elevated by a high salt diet). Recent studies have linked *α-adducin* to salt-sensitive hypertension but have not investigated the specific polymorphisms. Confirmation of the *α-adducin* G460W gene polymorphism as a mediator of salt-sensitive hypertension would define a high value target for hypertension therapy.

These association results are still controversial. An association between *α-adducin* G460W and EH has been demonstrated in Scandinavians and Japanese cohort [5]–[6], but no association was found in African-Americans, Australians of Britain ancestry, or Indians [7]–[9]. Liu et al (2010) performed a meta-analysis and failed to detect a genetic association between the *α-adducin* gene G460W polymorphism and hypertension in Caucasians, East Asians, South Africans, and African-Americans [10]. Furthermore, results from various population samples in China were also inconsistent. In 2011, Niu et al reported a null association between the *α-adducin* G460W gene polymorphism and hypertension in Chinese population in another meta-analysis [11]. In contrast, a meta-analysis of 20 stuides by Liu et al in 2011 suggested that the G460W polymorphism might increase the risk of hypertension in Chinese populations, especially in Han Chinese [12]. However, there were still some limitations in their work. For instance, the subgroup analysis by salt intake area was not performed. The literatures have not been retrieved completely. Two studies deviating from the Hardy-Weinberg equilibrium (HWE) were still included in that mata-analysis which inevitably influenced the results [13]–[14].

In consideration of these conflicting results and the above limitations, it is unclear yet whether there is a significant association between the *α-adducin* G460W gene polymorphism and EH in Chinese population. The current meta-analysis encompassing 23 studies with 5939 EH patients and 5021 controls was important to be performed to derive a more precise estimate of the association between *α-adducin* G460W gene polymorphism and EH in individual ethnicities within China. Compared with Liu's work, there were some similarities. For example, two statistical models as recessive and dominant genetic models were adopted in both meta-analysises. There were 18 individual studies overlapped between them. Because of the limitations in Liu's work, subgroup analysis stratified by salt intake was additionally conducted in the Chinese Han population. Moreover, the retrieved individual studies were more comprehensive and exact than those in Liu's work. So the present meta-analysis has potential to add to the current knowledge.

## Materials and Methods

### Publication search and inclusion criteria

The electronic databases PubMed, Embase, Web of Science, China Biological Medicine Database (CBMD), and China National Knowledge Infrastructure (CNKI) were searched for studies using the MeSH terms ‘hypertension’, ‘*α-adducin*’, ‘polymorphism’, ‘hypertension’, ‘gene’, and Chinese'. The compiled data set was last updated on May 4, 2011, and included published papers from 1999 to 2011.

The reports included had to meet the following criteria: (a) evaluation of the *α-adducin* G460W gene polymorphism and EH in a Chinese population, and (b) diagnosis of EH in agreement with the EH diagnosis criteria of the World Health Organization (WHO, 1999) where systolic blood pressure (BP)≥140 mmHg, diastolic BP≥90 mmHg, or treatment with antihypertensive medication defines EH.

### Data extraction

No paper was included if it did not meet the two aforementioned inclusion criteria. When the same study results appeared in several papers, only one study was used in this meta-analysis. The extracted data included the first author's name, publication date, region of study, ethnicity of the sample population, number of genotypes, and the total number of cases and controls (Supplement S1). Different ethnicities in China were categorized as Han, Kazakh, Mongolian, or She. The study regions included the three directly governed city regions Beijing, Shanghai, and Tianjin, the seven provinces Jiangsu, Fujian, Heilongjiang, Henan, Hebei, Shanxi, and Zhejiang, and the two autonomous regions Xinjiang, Neimenggu. These data were independently gathered using a standard retrieval protocol.

### Statistical analysis

Three statistical models as allelic genetic models (distribution of Trp allelic frequency of α-adducin gene), recessive genetic models (TrpTrp vs.GlyGly+GlyTrp) and dominant genetic models (GlyGly vs. GlyTrp+ TrpTrp) were used in the current meta-analysis.The odds ratio (OR) and corresponding 95% confidence interval (CI) was used to examine the association between the *α-adducin* G460W gene polymorphism and EH. The heterogeneity assumption was checked by the Chi-square-based Q-test and significance was set at P<0.05 [15]. The variation caused by the heterogeneity was also evaluated by calculating the inconsistency index *I^2^* where a higher value of *I^2^* indicates heterogeneity. A random-effects model was used for all analyses (the DerSimonian and Laird method) [16]–[17]. HWE was assessed by Fisher's exact test and P<0.05 indicated a significant deviation for HWE. Funnel plots were used to estimate the potential publication bias. The funnel plot asymmetry on the natural logarithm scale of the OR was assessed by Egger's linear regression test and significance was also set at P<0.05 [18]. Subgroup analysis stratified by salt intake was performed to observe the effect of dietary salt on the association of *α-adducin* G460W gene polymorphism and EH. Meta-regression was performed to explore the sources of heterogeneity. Significance was set at P<0.05. The statistical analysis was performed using STATA 10.0 software (StataCorp, College Station, TX).

## Results

### Studies and populations

**Figure 1:**
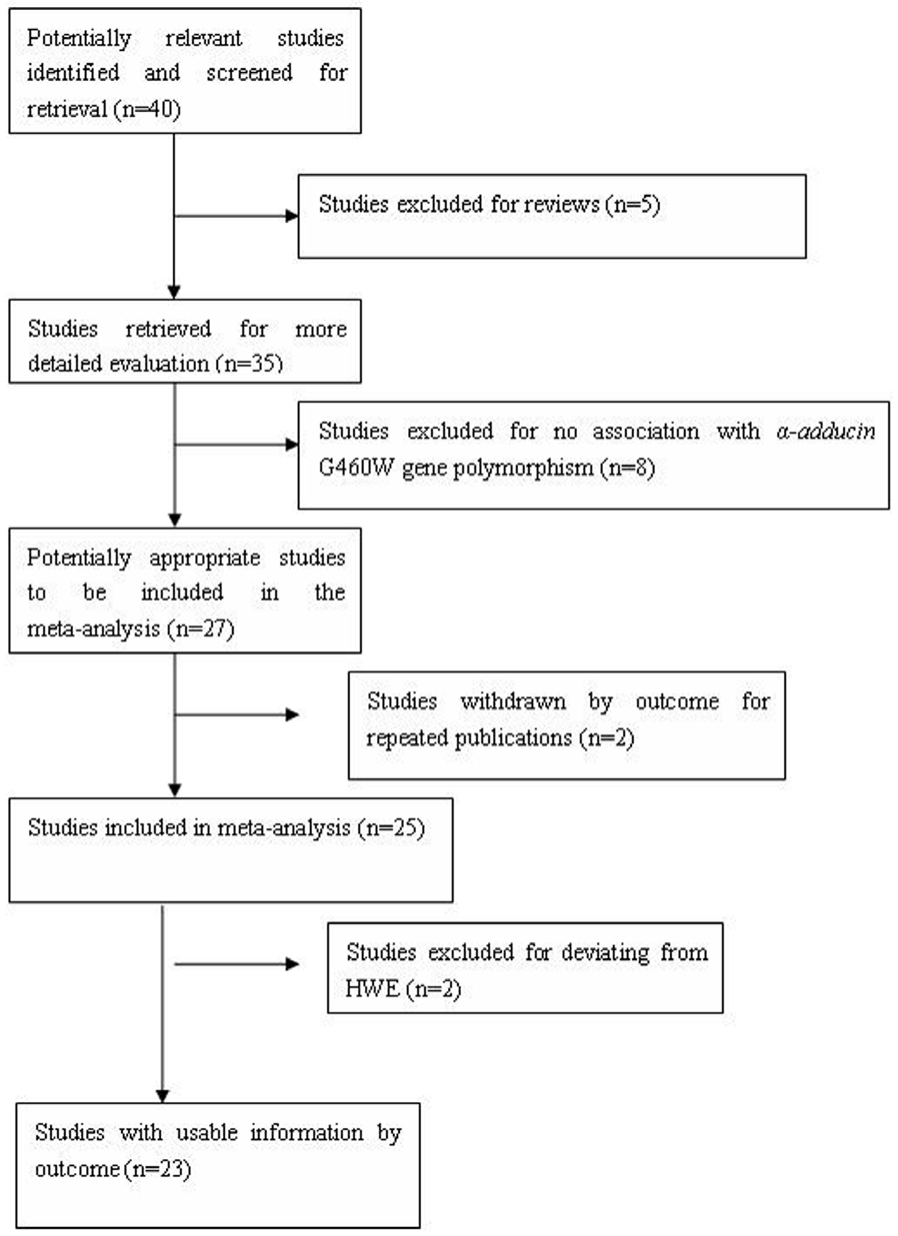
Flow diagram of articles selection process for *α-adducin* G460W gene polymorphism and EH risk meta-analysis.

Forty papers were retrieved by literature search, of which 23 conformed to the study inclusion criteria. Of the 17 excluded studies, 2 papers were published repeatedly, 5 papers were reviews, 2 deviated from HWE, and the remaining 8 did not focus on the G460W gene polymorphism (Figure 1). The total data of the 23 studies comprised 5939 EH patients and 5021 controls from 12 districts and 4 ethnicities, Han, Kazakh, Mongolian and She (Supplement S1) [19]–[41]. Eighteen studies with 5087 EH patients and 4183 controls were included in the Han subgroup. Three studies with 636 EH patients and 462 controls were included in the Kazakh subgroup. Only one study each focused on Mongolian and She populations, with 100 EH patients and 50 controls in the Mongolian subgroup and 116 EH patients and 326 controls in the She subgroup.

### Pooled analyses

**Figure 2:**
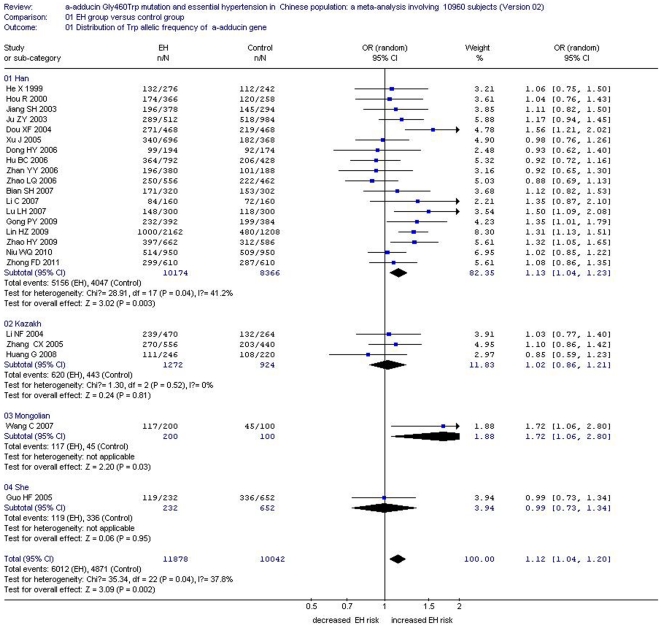
Forest plot of essential hypertension associated with *α-adducin* Gly460Trp gene polymorphism under an allelic genetic model (distribution of Trp allelic frequency of α-adducin gene) stratified by ethnicity.

**Figure 3:**
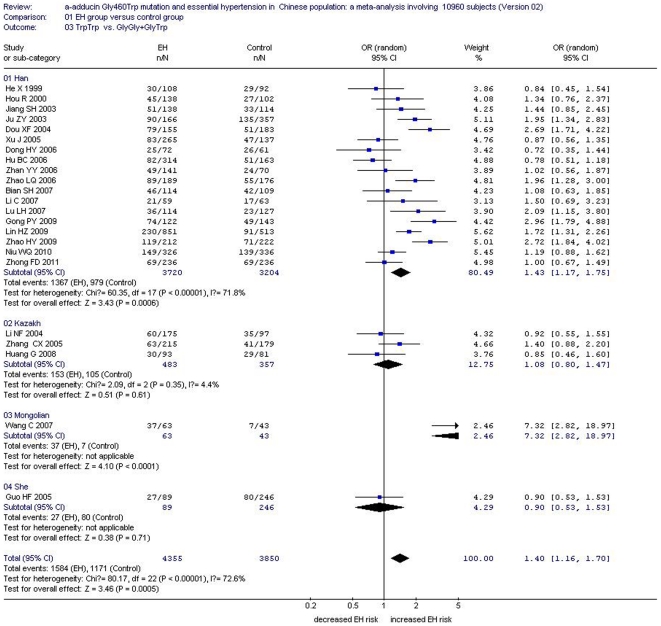
Forest plot of essential hypertension associated with *α-adducin* Gly460Trp gene polymorphism under a recessive genetic model (TrpTrp vs.GlyGly+GlyTrp) stratified by ethnicity.

**Figure 4:**
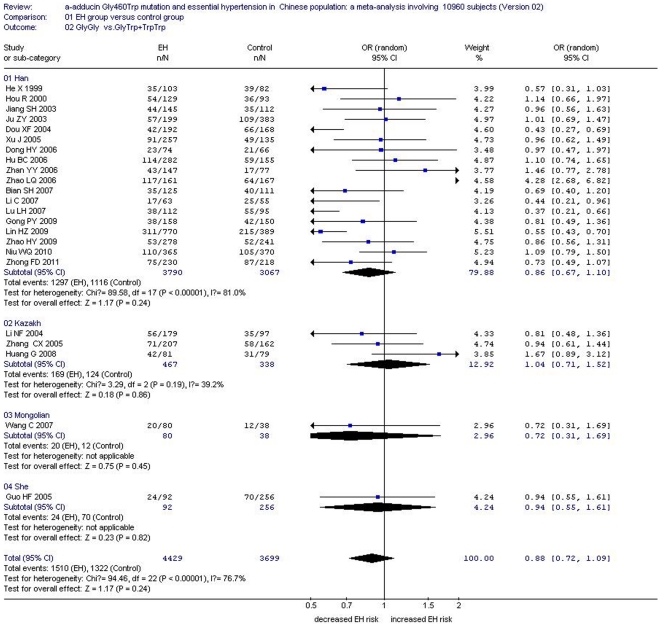
Forest plot of essential hypertension associated with *α-adducin* Gly460Trp gene polymorphism under a dominant genetic model (GlyGly vs. GlyTrp+ TrpTrp) stratified by ethnicity.

In the total Chinese population, there was a significant association between the *α-adducin* G460W gene polymorphism and EH under an allelic genetic model (OR: 1.12, 95% CI: 1.04–1.20, P = 0.002) and a recessive genetic model (OR: 1.40, 95% CI: 1.16–1.70, P = 0.0005). No significant association between the α-adducin Gly460Trp gene polymorphism and EH was observed in the dominant genetic model (OR: 0.88, 95% CI: 0.72–1.09, P = 0.24). In the stratified analysis, significantly increased risk was detected in the Han subgroup under an allelic genetic model (OR: 1.13, 95% CI: 1.04–1.23, P = 0.003) and a recessive genetic model (OR: 1.43, 95% CI: 1.17–1.75, P = 0.0006). (Supplement S2, Figures 2, 3 and 4)

The association between *α-adducin* G460W gene polymorphism and EH risk was significant in the Mongolian Chinese population under either the allelic genetic model (OR: 1.72, 95% CI: 1.06–2.80, P = 0.03) or recessive genetic model (OR: 7.32, 95% CI: 2.82–18.97, P<0.0001). There was no association evident in the Kazakh subgroup under an allelic genetic model (OR: 1.02, 95% CI: 0.86–1.21, P = 0.81) or a recessive genetic model (OR: 1.08, 95% CI: 0.80–1.47, P = 0.61). No significant association was found in the She subgroup under an allelic genetic model (OR: 0.99, 95% CI: 0.73–1.34, P = 0.95) or a recessive genetic model (OR: 0.90, 95% CI: 0.53–1.53, P = 0.71) (Supplement S2, Figures 2 and 3).

Under a dominant genetic model, no significant increased risk was found in the Han subgroup (OR: 0.86, 95% CI: 0.67–1.10, P = 0.24), Kazakh subgroup (OR: 1.04, 95% CI: 0.71–1.52, P = 0.86), Mongolian subgroup (OR: 0.72, 95% CI: 0.31–1.69, P = 0.45) or She subgroup (OR: 0.94, 95% CI: 0.55–1.61, P = 0.82) (Supplement S2, Figure 4)

**Figure 5:**
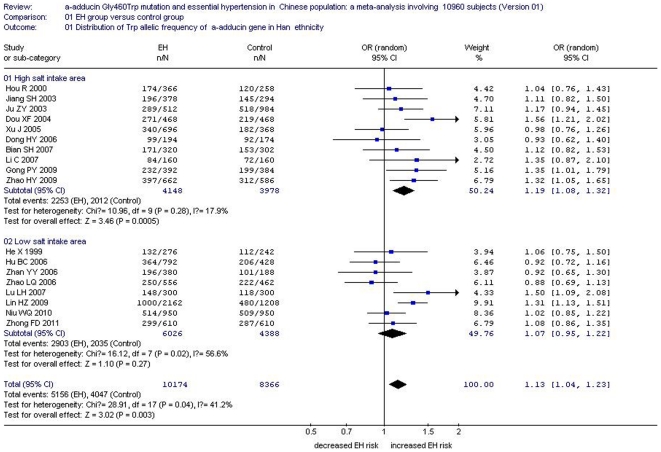
Forest plot of essential hypertension associated with *α-adducin* Gly460Trp gene polymorphism under an allelic genetic model (distribution of Trp allelic frequency of α-adducin gene) stratified by salt intake area in Chinese Han population.

**Figure 6:**
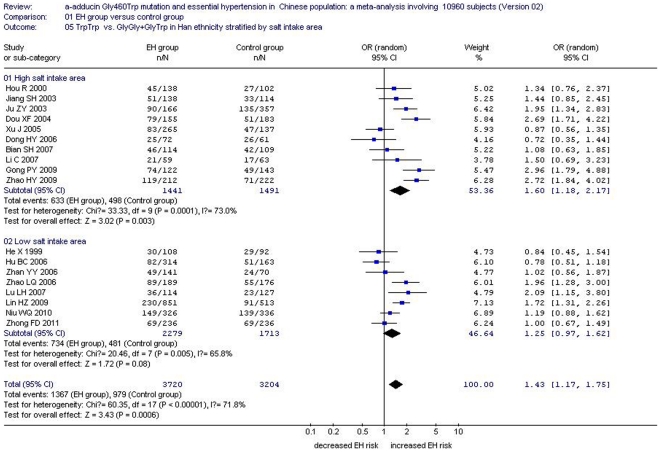
Forest plot of essential hypertension associated with *α-adducin* Gly460Trp gene polymorphism under a recessive genetic model (TrpTrp vs. GlyGly+GlyTrp) stratified by salt intake area in Chinese Han population.

**Figure 7:**
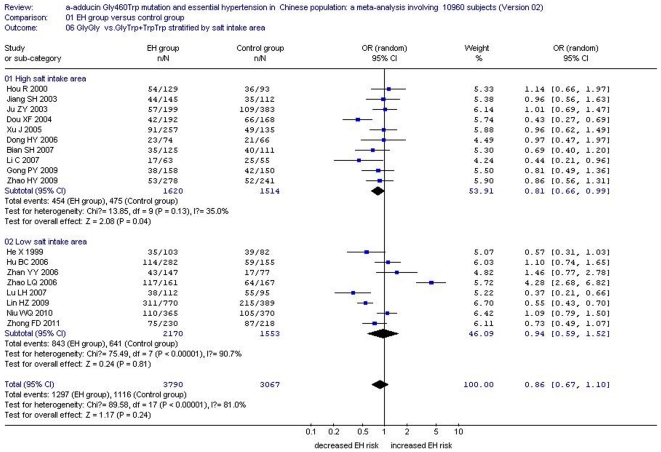
Forest plot of essential hypertension associated with *α-adducin* Gly460Trp gene polymorphism under a dominant genetic model (GlyGly vs. GlyTrp+ TrpTrp) stratified by salt intake area in Chinese Han population.

The relationship between the *α-adducin* G460W gene polymorphism and EH might be strongly affected by salt intake. Mean salt intake level was quite different between local areas and ethnic groups in China. Accordingly, subgroup analysis was stratified into a high salt intake area and low salt intake area according to provincial statistics for the Han subgroup. Southern China was referred to as the low salt intake area and northern China was considered the high salt intake area [42]. The two areas were divided by the Yangtze River. In the high salt intake area, there was a significant association under an allelic genetic model (OR: 1.19, 95% CI: 1.08–1.32, P = 0.0005) , a recessive genetic model (OR: 1.60, 95% CI: 1.18–2.17, P = 0.003) and a dominant genetic model (OR: 0.81, 95% CI: 0.66–0.99, P = 0.04). By contrast, no significant association between *α-adducin* G460W gene polymorphism and EH was found in the low salt intake area under an allelic genetic model (OR: 1.07, 95% CI: 0.95–1.22, P = 0.27), a recessive genetic model (OR: 1.25, 95% CI: 0.95–1.62, P = 0.08) or a dominant genetic model (OR: 0.94, 95% CI: 0.59–1.52, P = 0.81). (Supplement S2, Figures 5, 6 and 7)

Meta-regression was conducted to explore the potential sources of heterogeneity in the Han subgroup populations under an allelic genetic model of inheritance by using STATA 10.0 software. The study region, EH group sample size, control group sample size, total sample size, ratio of EH group sample size to control group sample size (RR), genotyping method were considered as the potential confounding factors. Among these factors, RR, genotyping method, total sample size and control group sample size might be possible explanations for the heterogeneity (*P* = 0.014, 0.032, 0.042, 0.046) (*P*<0.05), while EH group sample size was not associated (P>0.05) (Supplement S3 and S4).

**Figure 8:**
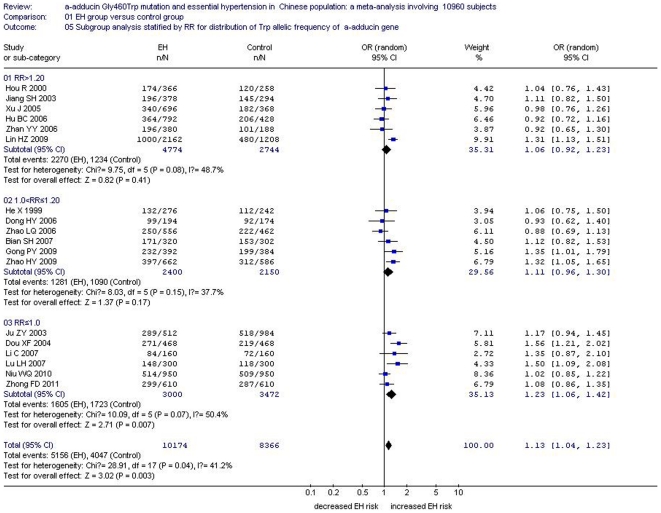
Forest plot of essential hypertension associated with *α-adducin* Gly460Trp gene polymorphism under an allelic genetic model (distribution of Trp allelic frequency of α-adducin gene) stratified by RR in Chinese Han population. RR: the ratio of case size to control size.

**Figure 9:**
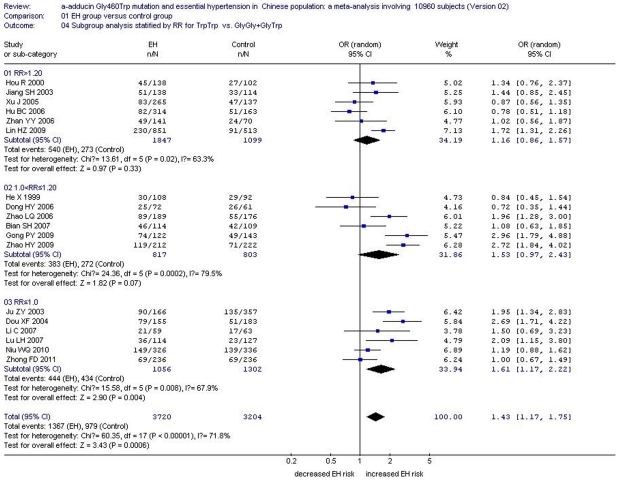
Forest plot of essential hypertension associated with *α-adducin* Gly460Trp gene polymorphism under a recessive genetic model (TrpTrp vs.GlyGly+GlyTrp) stratified by RR in Chinese Han population. RR: the ratio of case size to control size.

In the subgroup analysis stratified by RR under an allelic and a recessive model, subgroup 1 comprised studies with RR>1.20, while subgroup 2 included studies with 1.0<RR≤1.20. The residual studies with RR≤1.0 were classified as subgroup 3. In subgroup 1, no significant association was observed between *α-adducin* G460W gene polymorphism and EH under an allelic genetic model (OR: 1.06, 95% CI: 0.92–1.23, *P*_heterogeneity_ = 0.08, *I^2^* = 48.7%, *P* = 0.41) or under a recessive genetic model (OR: 1.16, 95% CI: 0.86–1.57, *P*_heterogeneity_ = 0.02, *I^2^* = 63.3%, *P* = 0.33). Similarly in subgroup 2, there was no significant association between *α-adducin* G460W gene polymorphism and EH under an allelic genetic model (OR: 1.11, 95% CI: 0.96–1.30, *P*_heterogeneity_ = 0.15, *I^2^* = 37.7%, *P* = 0.17) or under a recessive genetic model (OR: 1.53, 95% CI: 0.97–2.43, *P*_heterogeneity_ = 0.0002, *I^2^* = 79.5%, *P* = 0.07). However, in subgroup 3, a significant association was observed between *α-adducin* G460W gene polymorphism and EH under an allelic genetic model (OR: 1.23, 95% CI: 1.06–1.42, *P*_heterogeneity_ = 0.07, *I^2^* = 50.4%, *P* = 0.007) and under a recessive genetic model (OR: 1.61, 95% CI: 1.17–2.22, *P*_heterogeneity_ = 0.008, *I^2^* = 67.9%, *P* = 0.004). (Supplement S2, Figures 8, 9).

### Bias diagnostics

**Figure 10:**
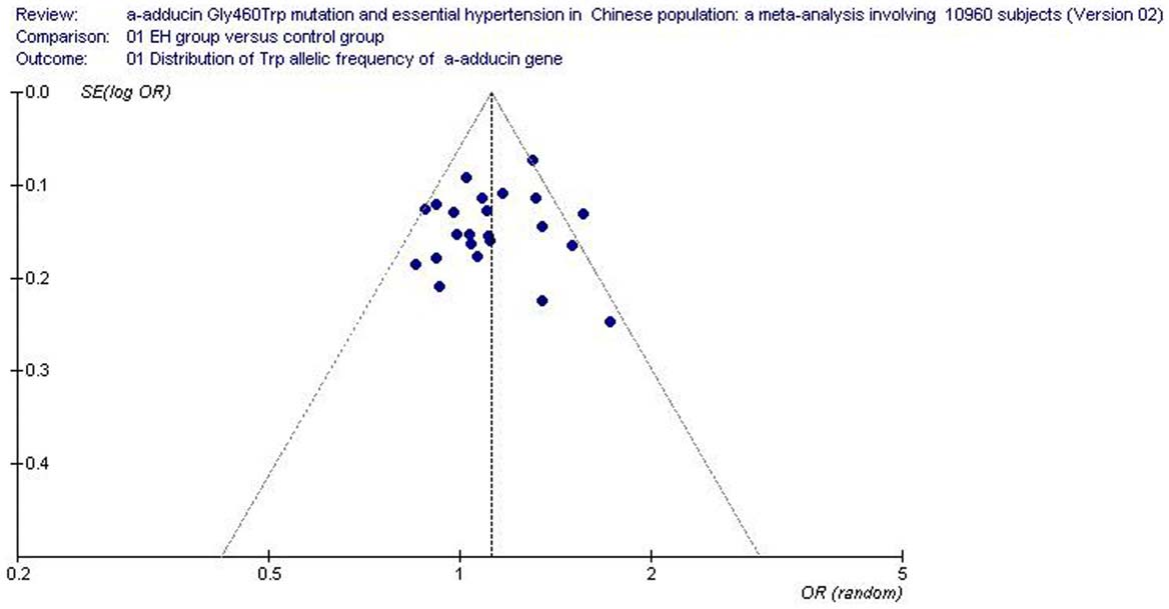
Funnel plot for studies of the association of essential hypertension and *α-adducin* Gly460Trp gene polymorphism under an allelic genetic model (distribution of Trp allelic frequency of α-adducin gene). The horizontal and vertical axis correspond to the OR and confidence limits. OR: odds ratio; SE: standard error.

The funnel plot and Egger's test were used to assess the publication bias of the literature for the allelic genetic model. No evidence of publication bias was revealed by the funnel plot (Figure 10). No significant difference was found in the Egger's test (T = 0.65, P = 0.523), which suggested that publication bias did not exist in the current meta-analysis.

## Discussion

In the current meta-analysis of 5,939 EH and 5,021 control Chinese subjects, the *α-adducin* G460W gene polymorphism was significantly associated with EH susceptibility under both the allelic (OR: 1.12) and recessive genetic model (OR: 1.40), while no association was found using a dominant genetic model (OR: 0.88). However, in the subgroup analysis by salt intake area, a significant association was found under the allelic (OR: 1.19), recessive genetic model (OR: 1.60) and dominant genetic model (OR: 0.81) in the high salt intake area subgroup of Chinese Han population.

In China, EH increases gradually after 30 years of age and is as high as 18.8% in the total Chinese population. Among the EH patients, about 60% are classified as salt-sensitive. Salt-sensitive hypertension is controlled by genetics, but exacerbated by dietary habits. The ratio of salt-sensitive hypertensive to normotensives ranges from 15% to 42%, while the ratio of salt-sensitive hypertensives to all hypertensives ranges from 28% to 74% in different regions of China. Indeed, it is 28.57% in southern China and 58% in northern China [31], [43]. Moreover, the salt-sensitivity increases with the age, especially in hypertensive patients [44].

Allelic variance of the *α-adducin* gene could influence Na, K-ATPase activity, thereby regulating water and sodium metabolism and affecting BP [45]–[46]. In 2009, Stenström reported that blocking the salt-inducible kinase 1 network prevented the increase in cell sodium transport caused by G460W, a hypertension-linked mutation in human *α-adducin* [47]. Hence, *α-adducin* is clearly a critical target for hypertension treatment in China. Not unexpectedly, the question of whether there is an association between the *α-adducin* G460W gene polymorphism and EH in Chinese adults has received a great deal of attention.

Differences in life styles might influence the role of the gene. For instance, the genetic effects were clearly different between the different salt intake areas [46]. The α-adducin protein plays a key role in the regulation of the water and salt metabolism. There was a significant difference in the salt intake level between the southern and northern Han Chinese population [42]. In the subsequent subgroup analysis by salt intake level, a significant association was found between the *α-adducin* G460W gene polymorphism and EH in the high salt intake area under the three genetic models, while no significant association was detected in the low salt intake area under the three genetic models. This result suggested that salt intake level actually had an effect on the relationship between the *α-adducin* G460W gene polymorphism and EH. Additionally, the higher OR in Mongolians might also reflect specific life-style factors like high salt intake (OR: 1.72 vs. 1.13, allelic genetic model; OR: 7.32 vs.1.43, recessive genetic model; OR: 0.72 vs. 0.86, dominant genetic model) [47].

In the meta-regression, the candidate confounding factors RR and genotyping methods were considered possible sources of heterogeneity. Indeed, the RR could partly explain the heterogeneity, indicating that the non-uniformity in the RR contributed to the between-study heterogeneity. The effect of genetics was greater in studies with RR≤1.0 than in other studies with RR>1.0, which suggested that the EH size and control size should be better balanced. After adjusting for RR, unexplained heterogeneity still existed. The association of the *α-adducin* G460W gene polymorphism and EH was distinctly weakened but still just significant in subsection 3 with RR≤1.0. Therefore, further studies should match the sizes of the cases and control populations.

The present meta-analysis results were generally consistent with the previous study by Liu [11], but there were several differences. The stratified subgroup analysis by salt intake level was only performed in the current study. Second, the She subgroup was not included in their work [41] and four studies of the Han population were excluded [19], [22], [34], [36]. Two studies by Jing et al. and Huang et al. that deviated from HWE were not excluded from Liu's research and excluded in the current meta-analysis [13]–[14]. Moreover, the heterogeneity source detection and corresponding subgroup analysis were not performed. Hence, the conclusions reached in the current meta-analysis provided stronger evidence for a relationship between *α-adducin* Gly460Trp gene polymorphism and EH in China.

In summary, the current meta-analysis suggested that the Gly460Trp allele of the *α-adducin* gene contributed to the susceptibility of EH in Chinese populations, especially in Han and Mongolia Chinese. Given that only one study has examined this relationship in Mongolian and She populations, additional studies should be launched to verify and extend these findings.

## Supporting Information

Supplement S1 **Characteristics of the investigated studies of the association between the** ***α-adducin*** **G460W polymorphism and essential hypertension in the Chinese population.**
(DOC)

Supplement S2 **Summary of meta-analysis of association of** ***α-adducin*** **Gly460Trp gene polymorphism and EH risk in the Chinese population.**
(DOC)

Supplement S3 **The confounding factors for the potential sources of heterogeneity studied by meta-regression in Chinese Han population under an allelic genetic model.**
(DOC)

Supplement S4 **The meta-regression results among 18 studies in Chinese Han population under an allelic genetic model.**
(DOC)

## References

[1] Guyton AC (1990). The surprising kidney-fluid mechanism for pressure control–its infinite gain!. Hypertension.

[2] Tripodi G, Valtorta F, Torielli L, Chieregatti E, Salardi S (1996). Hypertension-associated point mutations in the adducin alpha and beta subunits affect actin cytoskeleton and ion transport.. J Clin Invest.

[3] Beeks E, van der Klauw MM, Kroon AA, Spiering W, Fuss-Lejeune MJ (2004). Alpha-adducin Gly460Trp polymorphism and renal hemodynamics in essential hypertension.. Hypertension.

[4] Cusi D, Barlassina C, Azzani T, Casari G, Citterio L (1997). Polymorphisms of alpha-adducin and salt sensitivity in patients with essential hypertension.. Lancet.

[5] Melander O, Bengtsson K, Orho-Melander M, Lindblad U, Forsblom C (2000). Role of the Gly460Trp polymorphism of the alpha-adducin gene in primary hypertension in Scandinavians.. J Hum Hypertens.

[6] Narita I, Goto S, Saito N, Song J, Ajiro J (2003). Interaction between ACE and ADD1 gene polymorphisms in the progression of IgA nephropathy in Japanese patients.. Hypertension.

[7] Tiago AD, Nkeh B, Candy GP, Badenhorst D, Defterios D (2001). Association study of eight candidate genes with renin status in mild-to-moderate hypertension in patients of African ancestry.. Cardiovasc J S Afr.

[8] Wang WY, Adams DJ, Glenn CL, Morris BJ (1999). The Gly460Trp variant of alpha-adducin is not associated with hypertension in white Anglo-Australians.. Am J Hypertens.

[9] Ramu P, Umamaheswaran G, Shewade DG, Swaminathan RP, Balachander J (2010). Gly460Trp polymorphism of the ADD1 gene and essential hypertension in an Indian population: A meta-analysis on hypertension risk.. Indian J Hum Genet.

[10] Liu K, Liu J, Huang Y, Liu Y, Lou Y (2010). Alpha-adducin Gly460Trp polymorphism and hypertension risk: a meta-analysis of 22 studies including 14303 cases and 15961 controls.. PLoS One.

[11] Niu W, Qi Y (2011). Association of α-adducin and G-protein β3 genetic polymorphisms with hypertension: a meta-analysis of Chinese populations.. PLoS One.

[12] Liu K, Liu Y, Liu J, Wang Z, Lou Y (2011). α-adducin Gly460Trp polymorphism and essential hypertension risk in Chinese: a meta-analysis.. Hypertens Res.

[13] Jing S, Sun N, Wang H, Ma Z (2006). Association of the a-adducin Gly460Trp polymorphism with essential hypertension and blood pressure-lowing response to valsartan hydrochlorothiazide.. The Chinese Journal of Clinical Pharmacology.

[14] Huang X, Sun K, Song Y, Zhang H, Yang Y (2007). Association of a-adducin gene and G-protein b3-subuint gene with essential hypertension in Chinese.. National Medical Journal of China.

[15] Cochran WG (1968). The effectiveness of adjustment by subclassification in removing bias in observational studies.. Biometrics.

[16] Mantel N, Haenszel W (1959). Statistical aspects of the analysis of data from retrospective studies of disease.. J Natl Cancer Inst.

[17] DerSimonian R, Laird N (1986). Meta-analysis in clinical trials.. Control Clin Trials.

[18] Egger M, Davey Smith G, Schneider M, Minder C (1997). Bias in meta-analysis detected by a simple, graphical test.. BMJ.

[19] He X, Chu SL, Jin L, Xiong MM, Wang GL (1999). The relationship between a- -adducin gene polymorphism and essential hypertension.. Chinese Journal of Hypertension.

[20] Hou R, Liu ZQ, Xue MZ, Wang YX, Ye T (2000). Is Gly460Trp variant of a-adducin associated with essential hypertension in the Hans of Chinese population.. Chinese Journal of Medical Genetics.

[21] Jiang SH, Li XL, Zhang HF, Wang ZZ, De W (2003). Polymorphism of alpha-adducin, angiotensin-converting enzyme (ACE) genes, environmental factors and essential hypertension.. Chinese Journal of Hypertension.

[22] Ju Z, Zhang H, Sun K, Song Y, Lu H (2003). Alpha-adducin gene polymorphism is associated with essential hypertension in Chinese: a case-control and family-based study.. J Hypertens.

[23] Dou XF, Sun K, Huang XH, Liu XY, Ju ZY (2004). Adducin and angiotensin converting enzyme polymorphisms in essential hypertension.. Chinese Circulation Journal.

[24] Xu J, Hua Q, Li DB, Liu RK, Yang Z (2005). The association of gene polymorphism of a-adducin gene Gly460Trp with essential hypertension in Han nationality patients in Beijing.. Journal of Medical Postgraduates.

[25] Dong HY, Li QR, Wang Q, Luo ZG (2006). Association analysis between genetic polymorphism of ADD1 gene and GNB3 gene and essential hypertension.. South China Journal of Cardiology.

[26] Hu BC, Chu SL, Wang JG, Wang GL, Gao PJ (2006). Single nucleotide polymorphisms of three candidate genes in essential hypertension.. Chin J Intern Med.

[27] Zhan Y (2006). Studies on Candidate Genes of Essential Hypertension and Antihypertensive Pharmacogenomics.

[28] Zhao LQ, Gao PJ, Zhu DL (2006). Relationship between combination effect of ACE and adducing genes and essential hypertension.. Chinese Journal of Cardiovascular Medicine.

[29] Bian SH, Geng Q, Yu MY, Zhang Y, Xie YT (2007). Relationship between a-adducin gene polymorphism and essential hypertension.. Journal of Clinical Cardiology.

[30] Li C, Dang Q, Mu H (2007). Association study between essential hypertension and a-adducin P460Trp mutation.. Int J Cardiovasc Dis.

[31] Lu LH, Chen H, Luo JW, Wu XY, Lin HZ (2007). α-adducin in salt-sensitive hypertension and renal injury.. Molecular cardiology of China.

[32] Gong PY, Shen GM, Peng HM, Luo Y, Shen Y (2009). Association of the polymorphisms of sodium transport related genes with essential hypertension.. Chinese Journal of Medical Genetics.

[33] Lin HZ, Chen H, Luo JW, Wu XY, Chen Y (2009). Association among ACE, CYP11B2 and a-adducin gene polymorphisms with pulse pressure.. Chinese Journal of Cardiovascular Rehabilitation Medicine.

[34] Zhao HY, Cao J, Zhou L, Wang B, Qiu CC (2009). Genetic analysis of a-adducin and GNB3 in essential hypertenison patient.. Basic & Clinical Medicine.

[35] Niu WQ, Zhang Y, Ji KD, Gao PJ, Zhu DL (2010). Lack of association between alpha-adducin G460W polymorphism and hypertension: evidence from a case-control study and a meta-analysis.. J Hum Hypertens.

[36] Zhong FD, Zhang LN, Xu J, Zhang YM, Fei LJ (2011). Association of a-adducin gene polymorphisms with the risk of essential hypertension.. Basic & Clinical Medicine.

[37] Li NF, Li HJ, Zhou L, Nu EGL, Ouyang WJ (2004). The relationship between the a-adducin gene variant and essential hypertension in Kazakans of Xinjiang.. Science Technology and Engineering.

[38] Zhang CX, Liu Y, Lin RY, Wang SZ, Wang XF (2005). Connection between ADD1 gene polymorphism and essential hypertension from Xinjiang Kazakhs.. Fudan University Journal of Medical Sciences.

[39] Huang G, Wu P, Zhou T, Deng FM (2008). The relationship between a-adducin gene mutation and essential hypertension in Hazakh people of Shihezi district in Xinjiang Uygur autonomous region.. Journal of Shihezi University (Natural Science).

[40] Wang C, Sun G, Yan XL, Ding YC (2007). Study of a-adducin and endothelial nitric oxide synthase gene polymorphism in patients with essential hypertension in mongulia population.. Journal of Clinical Cardiology.

[41] Guo HF, Li Y, Wang GL, Lu YG, Zhou HF (2005). Association of peripheral and central blood pressure with the a-adducin Gly460Trp polymorphism in a Chinese population.. Chin J Cardiol.

[42] Liu LS, Gong LS (2005). Chinese Hypertension Prevention Guide revision Committee: Chinese Hypertension Prevention Guide 2005 Revision.

[43] Liu ZQ, Hou R, Liu J, Liu WH, Mou JJ (1998). Salt-sensitive Distribution in Population and the Characteristics of Salt Sensitive Subjects with Normotension.. Chinese Journal of Hypertension.

[44] Liu ZQ (2005). Salt-sensitivity of blood pressure and salt-sensitive hypertension.. Chin J Hypertension.

[45] Torielli L, Tivodar S, Montella RC, Iacone R, Padoani G (2008). alpha-Adducin mutations increase Na/K pump activity in renal cells by affecting constitutive endocytosis: implications for tubular Na reabsorption.. Am J Physiol Renal Physiol.

[46] Manunta P, Lavery G, Lanzani C, Braund PS, Simonini M (2008). Physiological interaction between alpha-adducin and WNK1-NEDD4L pathways on sodium-related blood pressure regulation.. Hypertension.

[47] Stenström K, Takemori H, Bianchi G, Katz AI, Bertorello AM (2009). Blocking the salt-inducible kinase 1 network prevents the increases in cell sodium transport caused by a hypertension-linked mutation in human alpha-adducin.. J Hypertens.

