# Supplementary material for: α-Adducin Gly460Trp Gene Mutation and Essential Hypertension in a Chinese Population: A Meta-Analysis including 10960 Subjects
Source: PLoS One. 2012 Jan 17;7(1):e30214. doi: 10.1371/journal.pone.0030214 (PMC3260257; doi:10.1371/journal.pone.0030214)
Supplement: Supplement S1 — Characteristics of the investigated studies of the association between the α-adducin G460W polymorphism and essential hypertension in the Chinese population. (DOC) [file pone.0030214.s001.doc]

**Supplement S1. Characteristics of the investigated studies of the association between the α-adducin G460W polymorphism and essential hypertension in the Chinese population**

| Author | Year | Region | Ethnicity | EH | | | Control | | | geno-typing | Study design | Matching criteria | sample size(EH/control) |
| --- | --- | --- | --- | --- | --- | --- | --- | --- | --- | --- | --- | --- | --- |
|  |  |  |  | GG | GW | WW | GG | GW | WW |  |  |  |  |
| He X[19] | 1999 | Shanghai | Han | 35 | 73 | 30 | 39 | 53 | 29 | MS-PCR | Case-control | Age,sex,ethnicity | 138/121 |
| Hou R[20] | 2000 | Shanxi | Han | 54 | 84 | 45 | 36 | 66 | 27 | PCR-SSCP | Case-control | Age,sex,ethnicity,BMI | 183/129 |
| Jiang SH [21] | 2003 | Jiangsu | Han | 44 | 94 | 51 | 35 | 79 | 33 | MS-PCR | Case-control | Ethnicity | 189/147 |
| Ju ZY [22] | 2003 | Beijing | Han | 57 | 109 | 90 | 109 | 248 | 135 | PCR-RFLP | Case-control | Age,sex,ethnicity,BMI | 256/492 |
| Dou XF[23] | 2004 | Beijing | Han | 42 | 113 | 79 | 66 | 117 | 51 | PCR-RFLP | Case-control | Age,sex,ethnicity | 234/234 |
| Xu J[24] | 2005 | Beijing | Han | 91 | 174 | 83 | 49 | 88 | 47 | MS-PCR | Case-control | Age,sex,ethnicity | 348/184 |
| Dong HY[25] | 2006 | Beijing | Han | 23 | 49 | 25 | 21 | 40 | 26 | MS-PCR | Case-control | Age,sex,ethnicity,BMI | 97/87 |
| Hu BC[26] | 2006 | Shanghai | Han | 114 | 200 | 82 | 59 | 104 | 51 | MS-PCR | Case-control | Age,sex,ethnicity | 396/214 |
| Zhan YY [27] | 2006 | Jiangsu | Han | 43 | 98 | 49 | 17 | 53 | 24 | PCR-RFLP | Case-control | Age, ethnicity | 190/94 |
| Zhao LQ[28] | 2006 | Shanghai | Han | 117 | 72 | 89 | 64 | 112 | 55 | MS-PCR | Case-control | Age,sex,ethnicity | 278/231 |
| Bian SH[29] | 2007 | Hebei | Han | 35 | 79 | 46 | 40 | 69 | 42 | PCR-RFLP | Case-control | Age,ethnicity | 160/151 |
| Li C [30] | 2007 | Tianjin | Han | 17 | 42 | 21 | 25 | 38 | 17 | PCR | Case-control | NA | 80/80 |
| Lu LH[31] | 2007 | Fujian | Han | 38 | 76 | 36 | 55 | 72 | 23 | PCR-RFLP | Case-control | Age,sex,ethnicity,BMI | 150/150 |
| Gong PY [32] | 2009 | Henan | Han | 38 | 84 | 74 | 42 | 101 | 49 | MS-PCR | Case-control | Age, ethnicity | 196/192 |
| Lin HZ[33] | 2009 | Fujian | Han | 311 | 540 | 230 | 215 | 298 | 91 | PCR-RFLP | Case-control | Age,sex,ethnicity | 1081/604 |
| Zhao HY[34] | 2009 | Heilongjiang | Han | 53 | 159 | 119 | 52 | 170 | 71 | PCR-RFLP | Case-control | Age,sex,ethnicity | 331/293 |
| Niu WQ[35] | 2010 | Shanghai | Han | 110 | 216 | 149 | 105 | 231 | 139 | PCR-RFLP | Case-control | Age,sex,ethnicity | 475/475 |
| Zhong FD[36] | 2011 | Zhejiang | Han | 75 | 161 | 69 | 87 | 149 | 69 | MS-PCR | Case-control | Age,sex,ethnicity | 305/305 |
| Li NF[37] | 2004 | Xinjiang | Kazakh | 56 | 119 | 60 | 35 | 62 | 35 | MS-PCR | Case-control | Age,sex,ethnicity | 235/132 |
| Zhang CX[38] | 2005 | Xinjiang | Kazakh | 71 | 144 | 63 | 58 | 121 | 41 | MS-PCR | Case-control | Age,sex,ethnicity,BMI | 278/220 |
| Huang G [39] | 2008 | Xinjiang | Kazakh | 42 | 51 | 30 | 31 | 50 | 29 | MS-PCR | Case-control | Age,sex,ethnicity | 123/110 |
| Wang C[40] | 2007 | Neimenggu | Mongolian | 20 | 43 | 37 | 12 | 31 | 7 | PCR-RFLP | Case-control | Age,sex,ethnicity | 100/50 |
| Guo HF[41] | 2005 | Zhejiang | She | 24 | 65 | 27 | 70 | 176 | 80 | PCR-RFLP | Case-control | Age,sex,ethnicity,BMI | 116/326 |

G460W: Gly460Trp

EH: essential hypertension

MS-PCR: mutagenically separated polymerase chain reaction;

PCR-RFLP: polymerase chain reaction-restriction fragment length polymorphism

PCR-SSCP: polymerase chain reaction-single-strand conformation polymorphism

NA: not applicable
