# Supplementary material for: α-Adducin Gly460Trp Gene Mutation and Essential Hypertension in a Chinese Population: A Meta-Analysis including 10960 Subjects
Source: PLoS One. 2012 Jan 17;7(1):e30214. doi: 10.1371/journal.pone.0030214 (PMC3260257; doi:10.1371/journal.pone.0030214)
Supplement: Supplement S2 — Summary of meta-analysis of association of α-adducin Gly460Trp gene polymorphism and EH risk in the Chinese population. (DOC) [file pone.0030214.s002.doc]

**Supplement S2. Summary of meta-analysis of association of α-adducin Gly460Trp gene polymorphism and EH risk in the Chinese population**

| **Genetic model** | **Ethnicity** | **Pooled OR (95% CI)** | **P value** | **Literature number** | **EH size** | **control size** | ***P*_heterogeneity_** |
| --- | --- | --- | --- | --- | --- | --- | --- |
| **allelic genetic model** | Han | 1.13(1.04-1.23) | 0.003^﹡^ | 18 | 5087 | 4183 | 0.04^﹡^ |
|  | Han: high salt intake area | 1.19(1.08-1.32) | 0.0005^﹡^ | 10 | 2074 | 1989 | 0.28 |
|  | Han: low salt intake area | 1.07(0.95-1.22) | 0.27 | 8 | 3013 | 2194 | 0.04^﹡^ |
|  | Han: subgroup1:RR>1.20 | 1.06(0.92-1.23) | 0.41 | 6 | 2387 | 1372 | 0.08 |
|  | Han: subgroup 2:1.0<RR≤1.2 | 1.11(0.96-1.30) | 0.17 | 6 | 1200 | 1075 | 0.15 |
|  | Han: subgroup 3: RR≤1.0 | 1.23(1.06-1.42) | 0.007^﹡^ | 6 | 1500 | 1736 | 0.07 |
|  | Kazakh | 1.02(0.86-1.21) | 0.81 | 3 | 636 | 462 | 0.52 |
|  | Mongolian | 1.72(0.06-2.80) | 0.03^﹡^ | 1 | 100 | 50 | NA |
|  | She | 0.99(0.73-1.34) | 0.95 | 1 | 116 | 326 | NA |
|  | **whole population** | **1.12(1.04-1.20)** | **0.002**^﹡^ | **23** | **5939** | **5021** | **0.04**^﹡^ |
| **recessive genetic model** | Han | 1.43(1.17-1.75) | 0.0006^﹡^ | 18 | 5087 | 4183 | <0.00001^﹡^ |
|  | Han: high salt intake area | 1.60(1.18-2.17) | 0.003^﹡^ | 10 | 2074 | 1989 | 0.0001 |
|  | Han: low salt intake area | 1.25(0.97-1.62) | 0.08 | 8 | 3013 | 2194 | 0.005 |
|  | Han: subgroup1:RR>1.20 | 1.16(0.86-1.57) | 0.33 | 6 | 2387 | 1372 | 0.02^﹡^ |
|  | Han: subgroup 2:1.0<RR≤1.2 | 1.53(0.97-2.43) | 0.07^﹡^ | 6 | 1200 | 1075 | 0.0002^﹡^ |
|  | Han: subgroup 3: RR≤1.0 | 1.61(1.17-2.22) | 0.004^﹡^ | 6 | 1500 | 1736 | 0.008^﹡^ |
|  | Kazakh | 1.08(0.80-1.47) | 0.61 | 3 | 636 | 462 | 0.35 |
|  | Mongolian | 7.32(2.82-18.97) | <0.0001^﹡^ | 1 | 100 | 50 | NA |
|  | She | 0.90(0.53-1.53) | 0.71 | 1 | 116 | 326 | NA |
|  | **whole population** | **1.40(1.16-1.70)** | **0.0005**^﹡^ | **23** | **5939** | **5021** | **<0.00001**^﹡^ |
| **Dominant genetic model** | Han | 0.86(0.67-1.10) | 0.24 | 18 | 5087 | 4183 | <0.00001^﹡^ |
|  | Han: high salt intake area | 0.81(0.66-0.99) | 0.04^﹡^ | 10 | 2074 | 1989 | 0.13 |
|  | Han: low salt intake area | 0.94(0.59-1.52) | 0.81 | 8 | 3013 | 2194 | <0.00001^﹡^ |
|  | Kazakh | 1.04(0.71-1.52) | 0.86 | 3 | 636 | 462 | 0.19 |
|  | Mongolian | 0.72(0.31-1.69) | 0.45 | 1 | 100 | 50 | NA |
|  | She | 0.94(0.55-1.61) | 0.82 | 1 | 116 | 326 | NA |
|  | **whole population** | **0.88(0.72-1.09)** | **0.24** | **23** | **5939** | **5021** | **<0.00001**^﹡^ |

**﹡P<0.05.**

**Abbreviations: CI:confidence interval; OR:odds ratio;EH size: the total number of EH cases; control size: the total number of control group.**
