# Supplementary material for: α-Adducin Gly460Trp Gene Mutation and Essential Hypertension in a Chinese Population: A Meta-Analysis including 10960 Subjects
Source: PLoS One. 2012 Jan 17;7(1):e30214. doi: 10.1371/journal.pone.0030214 (PMC3260257; doi:10.1371/journal.pone.0030214)
Supplement: Supplement S3 — The confounding factors for the potential sources of heterogeneity studied by meta-regression in Chinese Han population under an allelic genetic model. (DOC) [file pone.0030214.s003.doc]

**Supplement S3. The confounding factors for the potential sources of heterogeneity** **studied by meta-regression** **in Chinese Han population under an allelic genetic model**

| **Study** | **Year** | **Region** | **Case size** | **Control size** | **Total size** | **RR** | **Geno-typing** | **OR** |
| --- | --- | --- | --- | --- | --- | --- | --- | --- |
| He X[19] | 1999 | 1 | 138 | 121 | 259 | 1.14 | 1 | 1.06 |
| Hou R[20] | 2000 | 2 | 183 | 129 | 312 | 1.42 | 2 | 1.04 |
| Jiang SH [21] | 2003 | 1 | 189 | 147 | 336 | 1.29 | 1 | 1.11 |
| Ju ZY [22] | 2003 | 2 | 256 | 492 | 748 | 0.52 | 3 | 1.17 |
| Dou XF[23] | 2004 | 2 | 234 | 234 | 468 | 1.00 | 3 | 1.56 |
| Xu J[24] | 2005 | 2 | 348 | 184 | 532 | 1.89 | 1 | 0.98 |
| Dong HY[25] | 2006 | 2 | 97 | 87 | 184 | 1.11 | 1 | 0.93 |
| Hu BC[26] | 2006 | 1 | 396 | 214 | 610 | 1.85 | 1 | 0.92 |
| Zhan YY [27] | 2006 | 1 | 190 | 94 | 284 | 2.02 | 3 | 0.92 |
| Zhao LQ[28] | 2006 | 1 | 278 | 231 | 509 | 1.20 | 1 | 0.88 |
| Bian SH[29] | 2007 | 2 | 160 | 151 | 311 | 1.06 | 3 | 1.12 |
| Li C [30] | 2007 | 2 | 80 | 80 | 160 | 1.00 | 4 | 1.35 |
| Lu LH[31] | 2007 | 1 | 150 | 150 | 300 | 1.00 | 3 | 1.5 |
| Gong PY [32] | 2009 | 2 | 196 | 192 | 388 | 1.02 | 1 | 1.35 |
| Lin HZ[33] | 2009 | 1 | 1081 | 604 | 1685 | 1.79 | 3 | 1.31 |
| Zhao HY[34] | 2009 | 2 | 331 | 293 | 624 | 1.13 | 3 | 1.32 |
| Niu WQ[35] | 2010 | 1 | 475 | 475 | 950 | 1.00 | 3 | 1.02 |
| Zhong FD[36] | 2011 | 1 | 305 | 305 | 610 | 1.00 | 1 | 1.08 |

**Region 1: southern China; Region 2: northern China;**

**Case size: EH group sample size; Control size: Control group sample size; Total size: Total sample size;**

**RR: the ratio of case size to control size;**

**Geno-typing 1: MS-PCR; Geno-typing 2: PCR-SSCP; Geno-typing 3: PCR-RFLP; Geno-typing 4: PCR**

**LnOR: the natural logarithm of odds ratio for TrpTrp vs. GlyGly+GlyTrp between EH and control groups**
