# Supplementary material for: α-Adducin Gly460Trp Gene Mutation and Essential Hypertension in a Chinese Population: A Meta-Analysis including 10960 Subjects
Source: PLoS One. 2012 Jan 17;7(1):e30214. doi: 10.1371/journal.pone.0030214 (PMC3260257; doi:10.1371/journal.pone.0030214)
Supplement: Supplement S4 — The meta-regression results among 18 studies in Chinese Han population under an allelic genetic model. (DOC) [file pone.0030214.s004.doc]

**Supplement S4. The meta-regression results among 18 studies in Chinese Han population under an allelic genetic model**

|  | Coefficient | Standard Error | T value | P value | 95% Confidence Interval |
| --- | --- | --- | --- | --- | --- |
| control group sample size | -0.0023291 | 0.0010577 | -2.20 | 0.046^﹡^ | -0.0046141～-0.000044 |
| RR | -0.4349395 | 0.1526377 | -2.85 | 0.014^﹡^ | -0.7646932～-0.1051858 |
| total sample size | 0.0010132 | 0.0004494 | 2.25 | 0.042^﹡^ | 0.0000424 ～0.0019839 |
| Genotyping method | 0.0750853 | 0.0313625 | 2.39 | 0.032^﹡^ | 0.0073307 ～0.1428399 |
| cons | 0.5242675 | 0.2218294 | 2.36 | 0.034^﹡^ | 0.0450344～1.003501 |

﹡:P<0.05

RR: Ratio of EH and control group size;

Coefficient: regression coefficient.

The regression coefficients are the estimated increase in the lnOR per unit increase in the covariate as control sample size, ratio of EH and control group size, total sample size and genotyping method.

cons：constant item.
